# Detection and Management of Common Medication Errors in Internal Medicine Wards: Impact on Medication Costs and Patient Care

**DOI:** 10.15171/apb.2019.020

**Published:** 2019-02-21

**Authors:** Kamal Boostani, Hamid Noshad, Farahnoosh Farnood, Haleh Rezaee, Soheil Teimouri, Taher Entezari-Maleki, Reyhane Najafiazar, Azam Hassanpouri-Olia, Afshin Gharekhani

**Affiliations:** ^1^Drug Applied Research Center, Sina Hospital, Tabriz University of Medical Sciences, Tabriz, Iran.; ^2^Chronic Kidney Disease Research Center, Sina Hospital, Tabriz University of Medical Sciences, Tabriz, Iran.; ^3^Drug Applied Research Center, Department of Clinical Pharmacy (Pharmacotherapy), Faculty of Pharmacy, Tabriz University of Medical Sciences, Tabriz, Iran.; ^4^Department of Internal Medicine, Sina Hospital, Tabriz University of Medical Sciences, Tabriz, Iran.; ^5^Student Research Committee, Faculty of Pharmacy, Tabriz University of Medical Sciences, Tabriz, Iran.; ^6^Drug Applied Research Center, Department of Clinical Pharmacy (Pharmacotherapy), Sina Hospital, Tabriz University of Medical Sciences, Tabriz, Iran.

**Keywords:** Clinical pharmacist, Medication errors, Pharmaceutical care, Internal medicine

## Abstract

***Purpose:*** Medication errors (MEs) are a leading cause of morbidity and mortality, yet they have remained as confusing and underappreciated concept. The complex pharmacotherapy in hospitalized patients necessitates continued report and surveillance of MEs as well as persistent pharmaceutical care. This study evaluated the frequency, types, clinical significance, and costs of MEs in internal medicine wards.

***Methods:*** In this 8-month prospective and cross-sectional study, an attending clinical pharmacist visited the patients during each physician’s ward round at the morning. All MEs including prescription, transcription, and administration errors were detected, recorded, and subsequently appropriate corrective interventions were proposed during these rounds. The changes in the medications’ cost after implementing clinical pharmacist’s interventions were compared to the calculated medications’ cost, assuming that the MEs would not have been detected by clinical pharmacist and continued up to discharge time of the patients.

***Results: *** 89% of the patients experienced at least one ME during their hospitalization. A mean of 2.6 errors per patient or 0.2 errors per ordered medication occurred in this study. More than 70% of MEs happened at the prescription stage by treating physicians. The most prevalent prescription errors were inappropriate drug selection, unauthorized drugs and untreated indication. The highest MEs occurred on cardiovascular agents followed by antibiotics, and vitamins, minerals, and electrolytes. The net effect of clinical pharmacist’s contributions in medication therapy management was to decline medications’ costs by 33.9%.

***Conclusion:*** The role of clinical pharmacy services in detection, prevention and reducing the cost of MEs is of paramount importance to internal medicine wards.

## Introduction

Medication errors (MEs) are a leading cause of morbidity and mortality, yet they have remained as confusing and underappreciated concept.^1^ In health care, Institute of Medicine has defined an error as “the failure of a scheduled action to be completed as intended (error of execution) or the use of a wrong plan to attain an aim (error of planning). An error can be an act of commission or an act of omission.^2^ ME has been defined as “failure in the treatment process that leads to or has the potential to lead to harm to the patient”.^3,4^ MEs may occur at any stage of the medication use process including ordering, transcription, dispensing, administering, and monitoring.^3,4^ Wittich et aL^1^ noted that MEs are common and their type and frequency show marked variability by setting; they occur more frequently in intensive care units, for instance, where an average of 25 medications is administered for patients each day,^5^ and much less important in areas like obstetrics, where use of medications is generally prohibited. Previous studies have reported that MEs occur in 4.8% to 5.3% of hospitalized patients and result in 44 000 to 98 000 deaths in United States annually.^6-8^ It is known that MEs are highly prevalent among older patients or patients with multiple co-morbidities, various chronic diseases, and polypharmacy.^9-12^

Pharmaceutical care, or medication therapy management, has been a dynamic part of the health care system. Its major role is to guarantee appropriate use of medications so that patients’ health condition is improved.^13^ By having well-established patient care actions and services within the succeeded care setting, pharmacists are accredited to collaborate with other health care professionals to enhance quality of care provided to individual patients, maximize safety, improve outcomes, and decrease health care costs.^14^ MEs with multifactorial origins may cause suboptimal treatment. Also they have a potential of increasing morbidity and mortality in patients receiving medical or therapeutic services.^15^ Additionally, the patient’s confidence about health care system may be reduced and a high burden of cost may be imposed on the patients.^16^ Although clinical pharmacy services have been emerged from the past two decades in some teaching hospitals in Iran, their important role and services have not yet been represented in Northwest university teaching hospitals of Iran. So, we aimed to investigate the types, frequency, clinical significance, and burden of costs related to MEs in internal wards of Iranian Northwest university teaching hospital detected by clinical pharmacist.

## Materials and Methods

This was conducted as 8-month prospective and cross-sectional study from April 2016 at 20-bed internal ward of an academic university hospital affiliated to Tabriz University of Medical Sciences. One hundred adult patients who had at least one drug prescription during their hospitalization were randomly selected to enter into the study. Throughout the study, an attending clinical pharmacist, as an integral member of a health care team, visited the patients during each physician’s ward round at the morning. Any MEs were detected and subsequently appropriate corrective interventions were proposed during these rounds. All of the recommendations presented by the clinical pharmacist were rated as interventions that had the likelihood of acceptance or rejection by the physicians. MEs and clinical pharmacists’ interventions were appropriately collected using designed forms containing various information including demographic (age, gender, weight), clinical (chief complaint, diagnosis, past medical and medication history, drugs administered at internal wards, physical examination findings), and laboratory data. According to the coding system of Pharmaceutical Care Network European Foundation,^17^ MEs were categorized to 3 nodes of prescription, transcription, and administration. In prescription node, important MEs including improper drug, inappropriate dosage form, medication duplication, occurrence of any absolute contraindication, unauthorized drug (drug with no clear indication), omission error (no drug prescribing in spite of clear indication), administration of high or low drug’s dose or frequency, too high or short duration of therapy, and presence of drug interactions were detected and summarized. To identify MEs at transcription node, the congruence of physicians’ orders and nursery charts were evaluated by the clinical pharmacist. Administration errors were recorded as errors of drugs storage, preparation, dilution, and administration by nurses. Medication cost was described as the cost paid by insurance company and/or patient only for the drugs. The changes in the medications’ cost after implementing clinical pharmacist’s interventions were compared to the calculated medications’ cost, assuming that the MEs would not have been detected by clinical pharmacist and continued up to discharge time of the patients. The cost determined by this way, is only an estimate of the true cost. Some interventions of the clinical pharmacist increased medications’ cost while some others decreased related costs. Finally, the net effect of clinical pharmacist’s interventions on medications’ cost was evaluated based on the mathematical sum of the changes in medications’ regimen (US$1 was equal to 35 800 Iranian Rials during the study time). To evaluate clinical significance of MEs, one attending internist and one attending clinical pharmacist independently rated and categorized MEs using the guideline of the National Coordinating Council for Medication Error Reporting and Prevention (NCC MERP).^18^ To preclude biased judgments on the corrective interventions, at the time of disagreement between the two raters regarding the lower clinical significance of the intervention, the internist opinion on the significance of ME category was considered in decision making and analysis.

### Data analysis

Data analysis was performed using SPSS software (Statistical Package for the Social Sciences; version 17.0, Chicago, IL). Continuous and categorical data were presented as mean ± standard deviation (SD) or median (range) and number (percentage) as appropriate, respectively. Descriptive statistics were used to summarize frequency of MEs and direct related medications’ cost in this study. The rate of MEs was estimated by dividing the number of MEs by the number of patients and the number of ordered medications. Spearman rank correlation test was used to assess any correlations between the number of MEs and number of ordered drugs, age, and duration of hospital stay. *P* value < 0.05 was defined as statistically significant level.

## Results

During the study period, 100 patients (36 females and 64 males) with mean age of 63.5 ± 16.0 years old were randomly selected among 240 patients who had been admitted to internal medicine ward of our hospital during the 8 months. The total number of medications ordered to these patients was 1125. Two hundred thirty-three MEs on 89 patients were identified by attending clinical pharmacist. It means that 89% of the study patients experienced at least one ME during their hospitalization (Table 1). In other words, a mean of 2.6 errors per patient or 0.2 errors per ordered medication occurred in this study. Most MEs happened at the time of prescription by treating physicians (73.4% of errors) and the remaining was caused by nurses during transcription and drug administration (26.6% of errors).

**Table 1:** Patients’ demographic and clinical characteristics

**Variables**	
Age (y) (mean ± SD)	63.5 ± 16
Sex (female/male), No. (%)	36 (36)/64 (64)
Weight (kg) (mean ± SD)	66.6 ± 7.7
Height (cm) (mean ± SD)	163.5 ±5.1
Length of hospital stay (day) (mean)	7.4
Total number of ordered medications	1125
No. of ordered medications per patient, median (range)	10.5 (2-29)
No. of patients who experienced at least one ME	89
Total number of MEs	233
No. of MEs per patient, median (range)	2 (1-7)

The types and frequency of different MEs have been illustrated in Table 2. Interventions to correct MEs were provided by clinical pharmacist when they were detected. The acceptance rate of the clinical pharmacist’s interventions by treating physicians was 72.1% (168 out of 233 MEs). Rejection of the remaining clinical pharmacist’s interventions was mainly due to lack of ready and relevant information for rational pharmacotherapy decision-making or excessive nurses’ workload or missing imaging or laboratory data.

**Table 2:** Types and frequency of medication errors

**Medication error types**	**No.**	**%**
Prescription errors
Inappropriate drug (not most appropriate for indication)	34	14.6
Inappropriate dosage form (not most appropriate for indication)	4	1.7
Inappropriate duplication of therapeutic group or active ingredient	6	2.6
Contraindication for drug	4	1.7
No clear indication for drug use	30	12.9
No prescription of drug despite clear indication	24	10.3
Low drug dosage or frequency	15	6.4
High drug dosage or frequency	37	15.9
Too short treatment duration	2	0.9
Too long treatment duration	11	4.7
No control of drug interaction	4	1.7
Transcription errors	50	21.5
Administration errors
Drug not administered	9	3.9
Wrong drug administered	3	1.3

Table 3 summarizes several drug classes involved in MEs. The most common MEs occurred on cardiovascular drugs (26.6%) followed by antibiotics (21.9%), vitamins, minerals, and electrolytes (20.2%), and gastrointestinal drugs (13.7%). Total number of MEs demonstrated a significant and direct correlation with the total number of ordered medications (r = 0.48; *P* = 0.001) and patients’ length of in-hospital stay (r = 0.26; *P* = 0.007) but not with patients’ age (r = 0.044; *P* = 0.66). The mean number of MEs was not different between females and males (2.44 ± 1.63 vs. 2.25 ±1.52; *P* = 0.46) in our study.

**Table 3:** Distribution of medication errors among drug classes

**Drug classes**	**No. of errors**	**Percent of errors**
Cardiovascular drugs	62	26.6
Antibiotics	51	21.9
Vitamins, electrolytes, minerals	47	20.2
Gastrointestinal drugs	32	13.7
Others^a^	41	17.6

^a^Analgesics, anti-inflammatory, hormonal agents, psychiatric and neurologic drugs, pulmonary drugs, anti-histamines.

Categorization of MEs according to their clinical significance has been described in Table 4. Six MEs were differently categorized by the internist and clinical pharmacist. These errors were classified based on the internist’s opinion. As shown, none of the MEs caused the patients harm.

**Table 4:** Classification of medication errors based on their clinical significance (n = 233)

**Clinical significance**	**Definition** ^9^	**No. of errors (%)**
No error	Category A: circumstances for events that have the capacity to cause error	15 (6.5)
Error, No harm	Category B: An error occurred but the error did not reach the patient	34 (14.6)
	Category C: An error occurred that reached the patient but did not cause patient harm	138 (59.2)
	Category D: An error occurred that reached the patient and required monitoring to confirm that it resulted in no harm to the patient and/or required intervention to preclude harm	46 (19.7)
Error, Harm	Category E: An error occurred that may have contributed to or resulted in temporary harm to the patient and required intervention	0 (0)
	Category F: An error occurred that may have contributed to or resulted in temporary harm to the patient and required initial or prolonged hospitalization	0 (0)
	Category G: An error occurred that may have contributed to or resulted in permanent patient harm	0 (0)
	Category H: An error occurred that required intervention necessary to sustain life	0 (0)
Error, Death	An error occurred that may have contributed to or resulted in patient death	0

In this survey, after the clinical pharmacist’s interventions, total medication costs paid by the patients and their insurances reached $1450.8. Calculation of costs demonstrated that if MEs were not revised until the time of patients’ discharge, the total medications’ costs would have escalated by $746.5. Therefore, it is conceivable that clinical pharmacists’ interventions caused a decrease in patients’ medications’ costs by 33.9% ($7.46 per patient).

## Discussion

MEs are a leading cause of preventable morbidity and mortality among hospitalized patients.^8^ However, their importance is less considered in medical practice of Iran, and there have been only few researches performed in this field.^13,19-21^ So, further research on the prevalence of MEs and the role of clinical pharmacy services in detection and prevention of inappropriate drug use and patient’s harm is necessarily needed.

We found that 89% of the patients experienced at least one ME during their hospital stay which is lower than reported MEs rates by other studies in patients admitted to internal wards.^13^ This can be potentially explained by the different setting of visits in internal wards, number of properly educated and skilled nurses, variety of patients’ complaints, and absence of clinical pharmacists in clinical rounds or visits. In our study, a mean number of 2.6 errors per patient occurred which is lower than that reported from a nephrology ward of an Iranian teaching hospital (3.5 errors per patient).^20^ Different factors including poly-pharmacy, co-morbidities, and altered drugs’ pharmacokinetics may have contributed to higher frequency of MEs in kidney diseases patients. Most MEs (more than 70%) happened by the treating physicians at the prescription node and the remaining were errors at transcription and drug administration nodes caused by nurses. The most common types of prescription errors were inappropriate drug selection, unauthorized drugs and not prescribing drugs despite clear indication. The highest MEs were observed in cardiovascular agents followed by antibiotics, and vitamins, minerals, and electrolytes. These findings were consistent with the results of the previous researches in nephrology^20^ and internal wards of other hospitals.^13,19,21^ Obviously, high prevalence of cardiovascular co-morbid diseases, mineral and electrolytes disorders, and infectious diseases among patients admitted to internal medicine wards can logically justify the high rate of various drug use and consequently encountered MEs. Total number of MEs showed a marked correlation with the total number of ordered medications and patients’ length of hospitalization. Similar correlations were also reported in our previous study on patients with renal insufficiency admitted to nephrology ward,^20^ and in the study of Vazin et al^19^ performed on patients admitted to internal ICU. In spite of increased cost caused by some of the clinical pharmacist’s interventions in reducing MEs, the net effect of clinical pharmacist’s contributions in medication therapy management was to decline medications’ costs by 33.9%.

In a prospective observational study by Breuker et al^22^ which aimed to evaluate the prevalence, features, and severity of MEs and unintended medication discrepancies in an endocrinology-diabetology-nutrition department in France, 29.4% of the patients had at least one unintended medication discrepancy at admission or discharge and 98.2% of unintended medication discrepancies were considered as MEs. The average ME rate was 1.5 errors per patient at admission and 1.3 at discharge. As conceivable, prevalence of MEs is higher and cumulative error rate is comparable to our study. The most common MEs were omissions, wrong dose and frequency, and inappropriate added medications. The authors reported that 36% of patients experienced serious or very serious MEs and almost 40% potentially moderate MEs. In contrast to the study by Breuker et al,^22^ none of the MEs in our study resulted in harm to the patients. Additionally, in an observational study by Vazin et al^21^ on internal ICU patients in an Iranian teaching hospital, 89.4% of all MEs were categorized as not dangerous to the patients. However, one patient death occurred in this study due to the serious prescription MEs. Another study by Vazin et al in the same setting reported that 25.3% of MEs resulted in clinically significant harm to the ICU admitted patients.^19^ Diversity of these findings, along with higher rate of MEs in our survey, might be related to differences in patient’s care setting, definition and classification of MEs, and methods or sources of MEs detection.

Other studies reporting MEs in patients admitted to internal wards have been reviewed here. Noguchi et al^23^ conducted a prospective cohort study (JADE study) at 3 tertiary-care hospitals in japan to investigate epidemiology of MEs and adverse drug events in Japanese adult inpatients. They randomly selected 7 medical and 8 surgical wards among three included hospitals. 12.5% of the patients experienced MEs during their hospitalization. Most of the MEs (about 66%) happened at the ordering stage and the remaining respectively occurred at the monitoring (18.7%), administration (14%), and dispensing stages. The most prevalent MEs among all errors were duplicated drug orders (39%) followed by prescribing the incorrect frequency or dose (13%). A large majority of MEs by physicians happened at the prescribing stage (93.5%), whereas the MEs by nurses occurred respectively at the monitoring (48.4%) and administration stages (44.5%).^23^ Similarly, most of the MEs also occurred by physicians at the prescription node in our study. A 6-month Indian prospective interventional investigation was performed by Sinha et al^24^ in general medicine wards of a tertiary care hospital. ME rate was 6.4% and involvement of nurses in the happening of MEs was higher than treating physicians. Further, administration errors were more prevalent than prescription errors. They also reported that gastrointestinal drugs, particularly proton pump inhibitors, were the most involved drugs in the occurrence of MEs. Majority of the errors belonged to errors with no harm category. 90.6% of clinical pharmacists’ interventions were accepted by corresponding health-care professionals. Parallel results on the more involvement of nurses than treating physicians in the occurrence of MEs were also reported in studies by Acharya et al^25^ (67% vs. 33%) and Karna et al^26^ (61.6% vs. 16.1%). Moreover, the majority of the observed errors in their studies belonged to no harm category (96% and 89.8%, respectively). More prevalence of administration errors was also observed in the study of Karna et al.^26^ These findings were not consistent with those obtained in our study. This might be due to differences in the studies design, settings, definition and classification of MEs, source and way of MEs detection and reporting, number of educated health-care professionals and nursery workload in these studies in comparison with our one.

Prescription errors have been reported more frequently in Iranian studies that might be due to using paper-based traditional prescription system rather than professional computerized physician order entry, lack of available drug information references, and overlooked indication or dosage of medications.^22^ Majority of studies on MEs rate in patients admitted to internal wards reported higher rate of errors in stages of medication’s indication and dosage. Owing to the high prevalence of cardiovascular diseases among patients with internal problem, it would be expected that MEs occurring on cardiovascular drugs become more common.

Over the past years, hospital pharmacists’ clinical services have been provided to wards by performing daily ward visits and addressing any medication-related problems identified with proper interventions. The pharmacists’ role in prescribing stage has usually been retrospective that this may cause a long delay between the prescription time and their intervention, leading to increased risk of occurring costly adverse drug events. Thus, it seems more beneficial for pharmacists to attend at the time of prescription and provide specialized knowledge as it is needed.^27^ It has been demonstrated that participation of pharmacist in physician-led ward rounds along with daily ward visit yields markedly more interventions per patient compared to daily ward visit alone.^28^ Though time-consuming for pharmacists, attending daily physician-led ward rounds is a magnificent opportunity to preclude adverse drug events and decrease healthcare costs.^27-29^

Some important limitations of our study are summarized here. Our study was designed as a small cross-sectional investigation without including other hospitals’ internal wards divested of clinical pharmacy services. Our study assessed MEs only in internal medicine wards of one teaching hospital, rendering the extrapolation of the results to other wards or the same ward of other centers more difficult. Medication cost calculated in our study was an estimation of the exact cost.

## Conclusion

Majority of the patients admitted to internal medicine wards experienced MEs. MEs at the prescription node were the most frequent errors that occurred by treating physicians. The most prevalent types of prescription errors were inappropriate drug selection, unauthorized drugs and not prescribing drugs despite clear indication. The role of clinical pharmacy services in detection, prevention and reducing the cost of MEs is of paramount importance to internal medicine wards.

## Ethical Issues

This study obtained an ethics approval from local Ethics Committee of Tabriz University of Medical Sciences (XXX).

## Conflict of Interest

The authors declare that there is no conflict of interest.

## Acknowledgments

The authors do thank the nursery team of internal medicine ward of Sina Hospital of Tabriz University of Medical Sciences. The present study was a Pharm.D thesis performed at a teaching hospital of Tabriz University of Medical Sciences.
